# Effectiveness of vedolizumab dose escalation in inflammatory bowel disease in a large-scale, Canadian real-world cohort

**DOI:** 10.1093/jcag/gwaf033

**Published:** 2025-12-09

**Authors:** Edmond-Jean Bernard, Jean-Frederic Leblanc, A Hillary Steinhart, Abhinav Wadhwa, Marie-Julie Allard, Ryan Ward, Jessica Weiss, Christopher Pettengell, Brian Bressler

**Affiliations:** Department of Gastroenterology, Université de Montréal, Montréal, QC, H3T 1J4, Canada; Department of Gastroenterology, CHUM, Montréal, QC, H2X 0C1, Canada; Department of Gastroenterology, Université de Montréal, Montréal, QC, H3T 1J4, Canada; Department of Medicine, Sacre-Coeur Hospital, Montreal, QC, H4J 1C5, Canada; Department of Medicine, University of Toronto, Toronto, ON, M5S 3H2, Canada; Gastroenterology, Takeda Canada Inc, Toronto, ON, M5H 4E3, Canada; Gastroenterology, Takeda Canada Inc, Toronto, ON, M5H 4E3, Canada; Gastroenterology, Takeda Canada Inc, Toronto, ON, M5H 4E3, Canada; Clinical Department, Pentavere Research Group Inc, Toronto, ON, M6G 1A1, Canada; Clinical Department, Pentavere Research Group Inc, Toronto, ON, M6G 1A1, Canada; Division of Gastroenterology, University of British Columbia, Vancouver, BC, V6T 1Z7, Canada

**Keywords:** vedolizumab, epidemiology, real-world data, dose escalation

## Abstract

**Background and Aims:**

This study explores the effectiveness of vedolizumab dose escalation among patients with ulcerative colitis or Crohn’s disease who experienced a suboptimal or loss of clinical response in a Canadian real-world cohort.

**Methods:**

Patients with moderately to severely active ulcerative colitis or Crohn’s disease treated with vedolizumab were prospectively followed in a patient support program in Canada from 2015 to 2023. In patients who dose escalated to every 4 weeks from every 8 weeks intravenous maintenance dosing, Harvey-Bradshaw Index and Partial Mayo Scores were assessed 12 and 52 weeks after dose escalation. Clinical remission was defined as Harvey-Bradshaw Index < 5 or Partial Mayo Score < 3.

**Results:**

This study included 924 patients with Crohn’s disease (45% bio-naïve) and 1816 patients with ulcerative colitis (71% bio-naïve). Of patients with Crohn’s disease, 39% bio-naïve and 54% bio-experienced dose-escalated within the first 2 years. Of patients with ulcerative colitis, 39% bio-naïve and 50% bio-experienced dose escalated within the first 2 years. For Crohn’s disease patients receiving every 8 weekly intravenous maintenance dosing who were not in clinical remission, 50% bio-naïve and 23% bio-experienced patients were in clinical remission 12 weeks after dose escalation, while for ulcerative colitis, 43% bio-naïve and 35% bio-experienced patients were in clinical remission 12 weeks after dose escalation, which was sustained through 52 weeks.

**Conclusions:**

For patients who experienced a suboptimal or loss of clinical response to vedolizumab, this study supports the real-world effectiveness of intravenous vedolizumab dose escalation in improving clinical response and clinical remission rates among patients with ulcerative colitis or Crohn’s disease.

## Introduction

Inflammatory bowel disease (IBD) refers to a group of serious, chronic inflammatory conditions involving the gastrointestinal tract that affect more than 5 million people worldwide.^1^^,^^2^ Currently, the cause of both ulcerative colitis (UC) and Crohn’s disease (CD) is poorly understood. Due to the chronic nature of IBD, patients experience substantial negative effects on their quality of life, and it is associated with high rates of depression, fatigue, and anxiety.^3^^,^^4^ The primary goal of IBD treatment is to induce and maintain remission and avoid complications.^5^^,^^6^

Significant advances have been made in the treatment of these diseases, through the development of novel targeted treatments,^5-7^ and over the past few decades, monoclonal antibodies, including vedolizumab, have transformed IBD treatment.^7-9^ Vedolizumab is a gut-selective anti-lymphocyte trafficking drug indicated for the treatment of moderately to severely active CD and UC^7^ in adults, administered at a fixed dose (300 mg) by intravenous (IV) infusion at weeks 0, 2, and 6 during the induction period.^8^^,^^9^ Following treatment induction, patients can receive maintenance IV treatment of the same dose every 8 weeks (Q8W). Alternatively, patients can continue on subcutaneous maintenance vedolizumab by injection of 108 mg every 2 weeks (Q2W).^8^^,^^9^

For all available therapies, some patients experience a suboptimal response to treatment or a loss of response (LOR), after an initial response. In a systematic review and meta-analysis of clinical trials and observational studies, the pooled incidence rates of LOR to vedolizumab have been reported to be 47.9 for CD and 39.8 for UC per lifetime exposure, data primarily from patients previously treated with TNF antagonists.^10^

For patients who experience a LOR after induction therapy, dose escalation strategies are reported to be efficacious in recapturing response.^11-13^ Average rates of dose escalation of biologics within the first year of treatment are estimated to be 30% for CD and 36% for UC.^14^^,^^15^ As vedolizumab is provided as a fixed dose, intravenous dose escalation involves increasing the frequency of treatment to every 4 weeks (Q4W) per the Health Canada vedolizumab product monograph.^16^ In a recent systematic literature review including 10 real-world studies assessing the effectiveness of dose escalation of intravenous vedolizumab to Q4W during maintenance, it was reported that 49.6% of patients were able to recapture response.^10^^,^^13^ However, there is a lack of evidence on the real-world characteristics and outcomes of patients undergoing dose escalation within the Canadian context.

This was a retrospective study which leveraged data contained within the Takeda Canada patient support program (PSP) to generate real-world evidence (RWE) describing vedolizumab treatment, the effectiveness of intravenous dose escalation strategies, and clinical outcomes in a real-world setting.

## Materials and methods

### Study design

This was a retrospective, non-interventional cohort study using data collected as part of the Canadian Takeda PSP. All patients who received commercial vedolizumab between 2015 and 2023 in Canada were eligible to participate in this PSP. Included patients were ≥18 years at vedolizumab initiation, consented to secondary use of data, diagnosed with moderately to severely active CD or UC, and received vedolizumab IV 300 mg between the years 2015 and 2023.

Data including patient characteristics, disease history, vedolizumab use (including treatment start date, end date, infusion interval and dosage), and disease scores (Harvey-Bradshaw Index [HBI] for individuals with CD, and Partial Mayo Score [pMs] for those with UC) were recorded for treatment administration and/or reimbursement purposes and analyzed for this study.

### Outcomes

Clinical remission was defined as HBI <5, or pMs <3. Clinical response was defined as achieving clinical remission or an HBI decrease of ≥3 points from baseline/dose escalation, or a pMs decrease of ≥2 points and ≥25% from baseline/dose escalation.

Dose escalation was based on HCP assessment which may have included endoscopic assessment, biomarker results, and/or clinical assessment; however, the exact reason for vedolizumab dose escalation is not captured as a structured field in the PSP dataset and is not reported here. Clinical response/clinical remission was only explored among patients who were not in in HBI or pMs clinical remission at Q4W dose escalation, in order to understand the clinical effectiveness of dose escalation.

Patient characteristics, treatment patterns, clinical response, and clinical remission at treatment initiation and time to Q4W dose escalation were assessed for all patients with CD and UC, stratified by prior biologic exposure (bio-naïve or bio-exposed). Time to Q4W dose escalation was calculated using cumulative incidence curves. Time was measured by subtracting the date of first Q8W dose (at week 14) from the date of Q4W dose ­escalation; patients who did not dose escalate to Q4W were censored at their last Q8W dose. Stopping vedolizumab or changing to a different treatment schedule, including drug holidays, were treated as competing events.

Clinical response and clinical remission rates are reported 12 and 52 weeks following dose escalation to Q4W in order to understand the effectiveness of dose escalation in the presence of suboptimal clinical response or LOR. These outcomes were explored among patients who dose escalated to Q4W and were not in HBI or pMs clinical remission at Q4W dose escalation during the first 2 years of treatment, stratified by the type of dose escalation:

#### Q8W≥Q4W

This dose escalation cohort included adult patients with moderately to severely active UC or CD who initiated Q8W maintenance therapy, and were dose escalated to Q4W therapy any time after week 14.

#### W14≥Q4W

This dose escalation cohort included adult patients with moderately to severely active UC or CD who initiated Q4W at week 14.

#### W10≥Q4W

This dose escalation cohort included adult patients with moderately to severely active UC or CD who initiated Q4W at week 10.

Across dose escalation cohorts, results are also presented based on patients’ previous biologic exposure (bio-naïve or bio-experienced).

Time to clinical remission and clinical response from Q4W dose escalation were calculated for the 3 cohorts. This was calculated by subtracting the first Q4W date from the date of the first clinical response or clinical remission observed after Q4W. In cases of no clinical response/clinical remission, patients were censored at their date of last follow-up. In the competing risks model, stopping vedolizumab or changing to a different treatment schedule, including drug holidays, were treated as competing events.

Among patients who were primary clinical responders (in clinical response at week 14), association between clinical remission 12 weeks after Q4W dose escalation and patient characteristics (including diagnosis prior biologic exposure, timing of Q4W dose escalation, age, sex, baseline pMs/HBI, disease duration prior to vedolizumab initiation) was explored.

### Statistical analyses

HBI and pMs scores at specific time points (+/− 7 days) were used to determine baseline, dose escalation score, clinical response and clinical remission rates. When patients did not have data at the specific clinical response/clinical remission time point of interest, last observation carried forward method was applied. For post-induction analyses, only scores recorded after baseline are carried forward, and for analyses post dose escalation analyses, only scores recorded after escalation were carried forward.

Competing risk models were used for time to event analyses rather than Kaplan Meier curves, as they account for the possibility that different types of events (eg, stopping treatment), which might prevent the occurrence of the event of interest (dose escalation), thereby providing more accurate risk estimates in survival analysis.

Descriptive statistics were used to characterize the study population and cohorts. Continuous data are described as median and range, or mean and SD, depending on data distribution. Discrete data are described as frequencies and proportions. Time to events were analyzed using cumulative incidence curves. Numbers at risk are reported for each curve. Logistic regression was also used to determine association between patient characteristics and clinical remission (as measured by pMs/HBI scores) 12 weeks following Q4W dose escalation among patients not in clinical remission at Q4W dose escalation who were primary responders (in clinical response at week 14 and on Q8W).

### Ethical statement

This study was conducted with the highest respect for the individual participants in accordance with the requirements of the study protocol which was approved by Veritas Independent Review Board and also in accordance with: the ethical principles that have their origin in the Declaration of Helsinki; the International Conference on Harmonisation, E6 Good Clinical Practice: Consolidated Guideline; guidelines for good pharmacoepidemiology (GPP); and all applicable laws and regulations, including, without limitation, data privacy laws, clinical trial disclosure laws, and regulations, to protect the rights, safety, privacy, and well-being of study participants.

## Results

### Patient characteristics

The study cohort consisted of 924 (45% bio-naïve) patients with CD and 1816 patients (71% bio-naïve) with UC. The median (IQR) duration of follow-up was 18 months (10-29) for patients with CD and 17 months (9-29) for patients with UC (Table 1). The median (IQR) age was 48 years (37-60) for patients with CD and 44 years (32-61) for patients with UC. Of patients with CD and UC, 570 (62%) and 930 (51%) of patients were female, respectively (Table 1). Among patients with CD, the median (IQR) baseline HBI score was 10 (9-13), and for patients with UC the median (IQR) baseline pMs score was 6 (5-8) (Table 1). Disease duration prior to vedolizumab initiation was longer in bio-experienced patients versus bio-naïve: 15 versus 3 years among patients with CD and 6 versus 4 years among patients with UC (Table 1).

**Table 1.** Baseline demographics and clinical characteristics of included patients with CD or UC stratified by previous biologic exposure.

	Crohn’s disease		Ulcerative colitis	
	Bio-experienced (*N* = 511)	Bio-naïve (*N* = 413)	Bio-experienced (*N* = 522)	Bio-naïve (*N* = 1294)
**Sex**				
**Female**	314 (61%)	256 (62%)	269 (52%)	661 (51%)
**Male**	197 (39%)	156 (38%)	251 (48%)	631 (49%)
**Other**	0 (0%)	1 (0%)	2 (0%)	2 (0%)
**Age at vedolizumab initiation (years)**				
**Median**	47	50	43	45
**IQR**	35-58	38-61	31-60	32-61
**Disease duration**				
**Median**	15	3	6	4
**IQR**	7-26	0-18	2-12	1-11
**Missing**	43	69	53	163
**Follow-up (months)**				
**Median**	18	20	20	16
**IQR**	10-29	11-29	10-31	9-29
**Baseline HBI**				
**Median**	10	10	NA	NA
**IQR**	9-13	8-12	NA	NA
**Missing**	0	0	522	1294
**Baseline pMs**				
**Median**	NA	NA	6	6
**IQR**	NA	NA	5-7	5-8
**Missing**	511	413	0	0

Abbreviations: HBI, Harvey-Bradshaw Index; pMs, Partial Mayo Score;

### Clinical remission and clinical response following vedolizumab initiation

**Figure 1.**
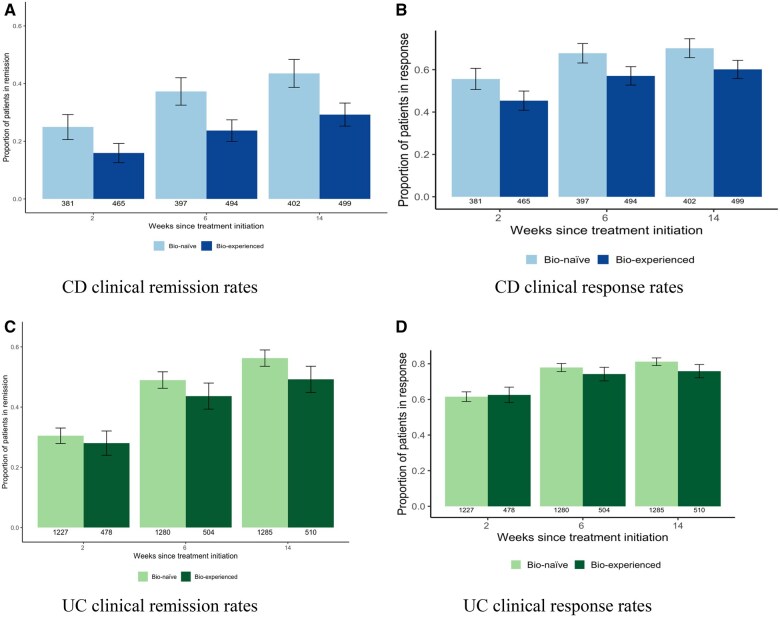
Clinical remission and clinical response rates at 2, 6, and 14 weeks following treatment initiation for included patients with CD and UC stratified by previous biologic exposure. (A) CD clinical remission rates. (B) CD clinical response rates. (C) UC clinical remission rates. (D) UC clinical response rates.

At 2, 6, and 14 weeks following treatment initiation, 169 (20%), 265 (30%), and 312 (36%), patients with CD experienced clinical remission, respectively (Figure 1A). For patients with UC, at 2, 6, and 14 weeks following treatment initiation, 508 (30%), 847 (47%), and 974 (54%) patients experienced clinical remission, respectively (Figure 1C). At 2, 6, and 14,weeks following treatment initiation, 423 (50%), 551 (62%), and 582 (65%) patients with CD experienced clinical response, respectively (Figure 1B). For patients with UC, at 2, 6, and 14 weeks following treatment initiation, 1054 (62%), 1371 (77%), and 1430 (80%, patients experienced clinical response, respectively (Figure 1D).

### Rates of Q4W dose escalation

Among patients with CD, 165/413 (39%) of bio-naïve patients and 273/511 (54%) of bio-experienced patients dose-escalated to Q4W within the first 2 years (Table 2). Among patients with UC, 489/1294 (39%) of bio-naïve patients and 258/522 (50%) of bio-experienced patients dose-escalated to Q4W within the first 2 years (Table 2).

**Table 2.** Treatment and dose escalation patterns of included patients with CD or UC stratified by previous biologic exposure.

Treatment/dose escalation pattern	Crohn’s disease		Ulcerative colitis	
	Bio-naïve (*N* = 413)	Bio-experienced (*N* = 511)	Bio-naïve (*N* = 1294)	Bio-experienced (*N* = 522)
**Initiated Q8W maintenance therapy, and were dose escalated to Q4W therapy after week 14 (Q8W>Q4W)**	97 (23%)	153 (30%)	255 (20%)	118 (23%)
**Initiated Q8W maintenance and received re-induction of therapy and dose escalated Q4W therapy thereafter**	1 (0%)	4 (1%)	7 (1%)	4 (1%)
**Dose escalated to Q4W therapy at week 14 (W14>Q4W)**	43 (10%)	56 (11%)	138 (11%)	74 (14%)
**Dose escalated to Q4W therapy at week 10 (W10>Q4W)**	24 (6%)	60 (12%)	89 (7%)	62 (12%)
**Initiated and stayed on Q8W maintenance therapy without dose escalation for 2 years**	59 (14%)	57 (11%)	216 (17%)	84 (16%)
**Initiated Q8W maintenance therapy, had less than 2 years of follow-up, and did not dose escalate during that time**	189 (46%)	181 (35%)	589 (46%)	180 (34%)

**Figure 2.**
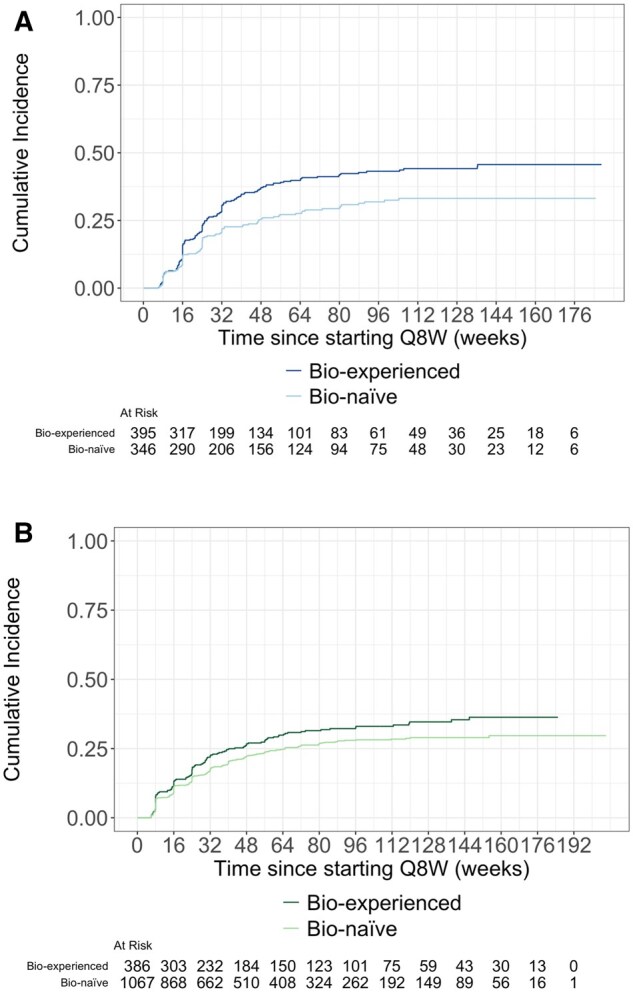
(A) Time to Q4W dose escalation for included patients starting at week 14 with CD stratified by previous biologic exposure. (B) Time to Q4W dose escalation for included patients starting at week 14 with UC stratified by previous biologic exposure.

Among patient cohorts which had not yet dose escalated by week 14, 97 (28%) of bio-naïve patients with CD and 153 (39%) bio-experienced patients with CD had dose escalated to Q4W 26 weeks after initiating Q8W treatment (Figure 2A). Among patients with UC, 255 (24%) of bio-naïve patients and 118 (31%) of bio-experienced patients had dose escalated to Q4W 26 weeks after starting Q8W (Figure 2B).

### Clinical response and clinical remission at dose escalation and at 12- and 52-week follow-up

Among patients who dose escalated to Q4W, 297 (68%) patients with CD (104 bio-naïve, 193 bio-experienced) were not in HBI clinical remission. Of bio-naïve patients, 55 (53%) patients Q8W>Q4W dose escalated, 34 (33%) W14>Q4W dose escalated, and 15 (14%) W10>Q4W dose escalated. Of bio-experienced patients, 108 (56%) patients Q8W>Q4W dose escalated, 42 (22%) W14>Q4W dose escalated, and 43 (22%) W10>Q4W dose escalated (Table 3).

**Table 3.** Proportion of patients in clinical remission and clinical response 12 and 52 weeks following Q4W dose escalation.

		Crohn’s disease						Ulcerative colitis					
		Bio-naïve			Bio-experienced			Bio-naïve			Bio-experienced		
		(*N* = 104)			(*N* = 193)			(*N* = 315)			(*N* = 166)		
		Q8W>	W14>	W10>	Q8W>	W14>	W10>	Q8W>	W14>	W10>	Q8W>	W14>	W10>
		Q4W	Q4W	Q4W	Q4W	Q4W	Q4W	Q4W	Q4W	Q4W	Q4W	Q4W	Q4W
		(*N* = 55)	(*N* = 34)	(*N* = 15)	(*N* = 108)	(*N* = 42)	(*N* = 43)	(*N* = 151)	(*N* = 95)	(*N* = 69)	(*N* = 70)	(*N* = 53)	(*N* = 43)
**Week 12**	In Clinical remission (%)	25 (50%)	3 (11%)	2 (17%)	24 (23%)	8 (22%)	9 (24%)	61 (43%)	27 (39%)	14 (28%)	23 (35%)	18 (40%)	7 (20%)
	In Clinical response (%)	32 (64%)	12 (43%)	4 (33%)	46 (45%)	20 (56%)	20 (54%)	79 (56%)	40 (58%)	25 (50%)	37 (57%)	25 (56%)	13 (37%)
	Missing	5	6	3	5	6	6	9	26	19	5	8	8
**Week 52**	In Clinical remission (%)	13 (52%)	6 (43%)	0 (0%)	19 (39%)	6 (33%)	7 (47%)	26 (68%)	10 (67%)	13 (68%)	19 (83%)	12 (57%)	13 (81%)
	In Clinical response (%)	13 (52%)	10 (71%)	1 (50%)	31 (63%)	11 (61%)	11 (73%)	33 (87%)	12 (80%)	16 (84%)	21 (91%)	14 (67%)	15 (94%)
	Missing	30	20	13	59	24	28	113	80	50	47	32	27

Among bio-naïve patients with CD, 25 (50%) patients in the Q8W>Q4W dose escalation cohort were in clinical remission 12 weeks following dose escalation (Table 3). In the W14>Q4W and W10>Q4W dose escalation cohorts, 3 (11%) and 2 (17%) patients were in clinical remission at 12 weeks following dose escalation (Table 3). Among bio-experienced patients with CD, 24 (23%) patients in the Q8W>Q4W dose escalation cohort were in clinical remission 12 weeks following dose escalation (Table 3). In the W14>Q4W and W10>Q4W dose escalation cohorts, 8 (22%) and 9 (24%) patients were in clinical remission at 12 weeks following dose escalation (Table 3). Results for bio-naïve and bio-experienced CD patients were sustained or improved through to 52 weeks following dose escalation (Table 3); however, patient numbers were low at week 52.

**Figure 3.**
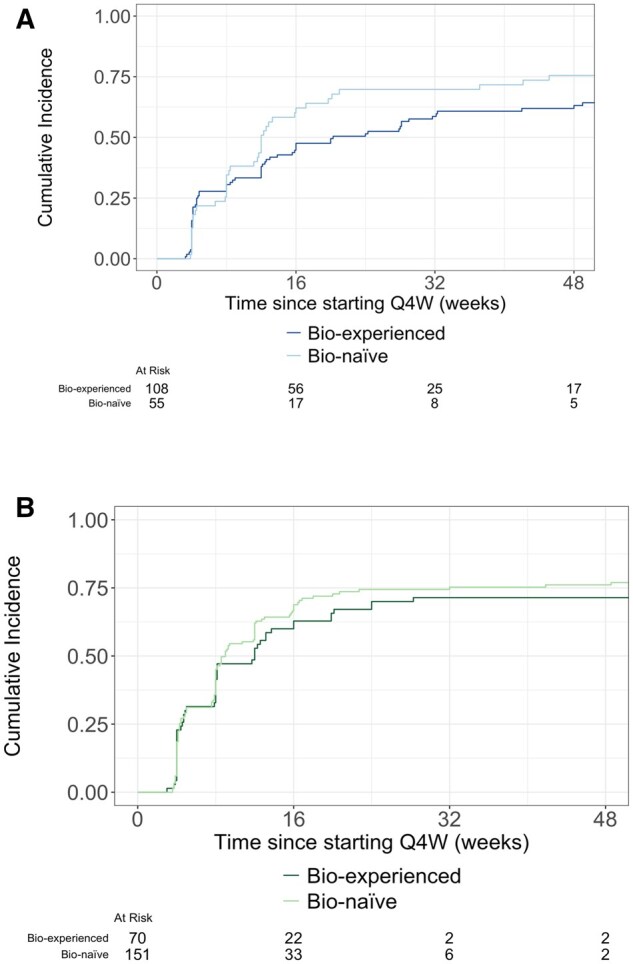
(A) Time to clinical remission following Q4W dose escalation among CD patients in the Q8W>Q4W dose escalation cohort. (B) Time to clinical remission following Q4W dose escalation among UC patients in the Q8W>Q4W dose escalation cohort.

**Figure 4.**
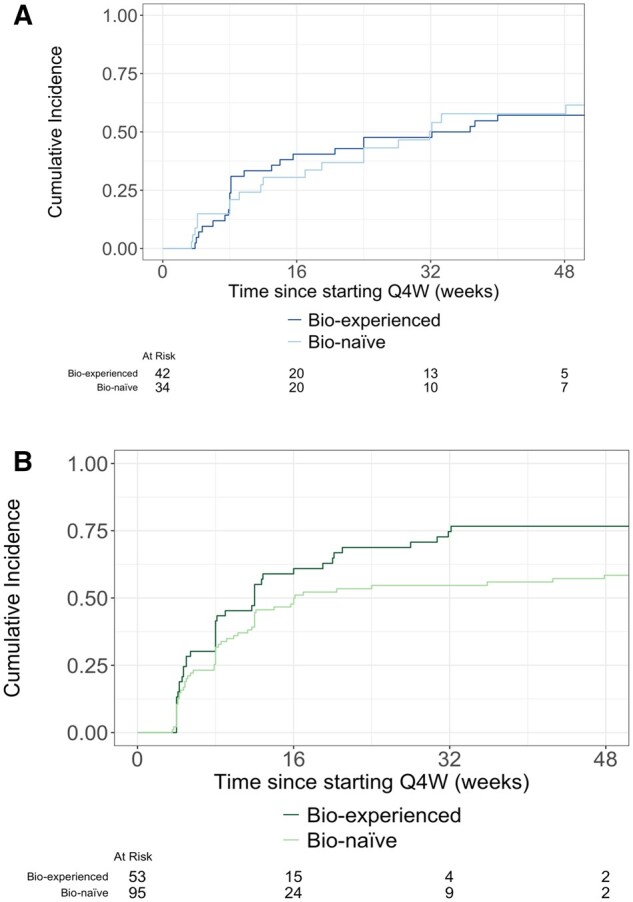
(A) Time to clinical remission following Q4W dose escalation among CD patients in the W14>Q4W dose escalation cohort. (B) Time to clinical remission following Q4W dose escalation among UC patients in the W14>Q4W dose escalation cohort.

**Figure 5.**
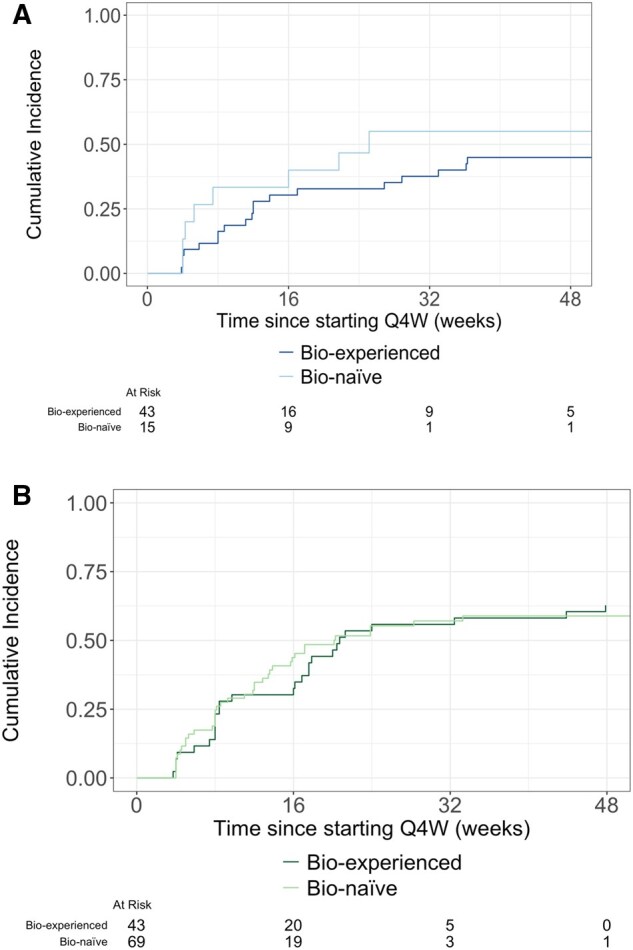
(A) Time to clinical remission following Q4W dose escalation among CD patients in the W10>Q4W dose escalation cohort. (B) Time to clinical remission following Q4W dose escalation among UC patients in the W10>Q4W dose escalation cohort.

Time to first clinical remission is displayed in Figures 3A, 4A, and 5A. Time to first clinical response is displayed in Figures S1A, S2A, and S3A.

Among patients who dose escalated to Q4W, 481 (64%) patients with UC (315 bio-naïve, 166 bio-experienced) were not in pMs clinical remission. Of bio-naïve patients, 151 (48%) patients Q8W>Q4W dose escalated, 95 (30%) W14>Q4W dose escalated, and 69 (22%) W10>Q4W dose escalated. Of bio-experienced patients, 70 (42%) patients Q8W>Q4W dose escalated, 53 (32%) W14>Q4W dose escalated, and 43 (26%) W10>Q4W dose escalated.

Among bio-naïve patients with UC, 61 (43%) patients in the Q8W>Q4W dose escalation cohort were in clinical remission at 12 weeks following dose escalation (Table 3). In the W14>Q4W and W10>Q4W dose escalation cohorts, 27 (39%) and 14 (28%) patients were in clinical remission at 12 weeks following dose escalation (Table 3). Among bio-experienced patients with UC, 23 (35%) patients in the Q8W>Q4W dose escalation cohort were in clinical remission 12 weeks following dose escalation (Table 3). In the W14>Q4W and W10>Q4W dose escalation cohorts, 18 (40%) and 7 (20%) patients were in clinical remission at 12 weeks following dose escalation (Table 3). Results for bio-naïve and bio-experienced UC patients were sustained or improved through to 52 weeks following dose escalation (Table 3); however, patient numbers were low at week 52.

Time to first clinical remission is displayed in Figures 3B, 4B, and 5B. Time to first clinical response is displayed in Figures S1B, S2B, and S3B. Clinical response rates 12 and 52 weeks following treatment initiation are presented in Table 3.

### Association between clinical remission and baseline characteristics

Logistic regression was used to assess for baseline characteristics associated with clinical remission including previous biologic, diagnosis, patient sex, age, disease duration prior to treatment, and baseline pMs/HBI scores were not found to be significant (Table S1).

## Discussion

This retrospective, non-interventional cohort analysis examined 2740 patients with CD and UC who received vedolizumab within a nationwide Canadian PSP. The study explored the effectiveness of intensification strategies among individuals experiencing suboptimal clinical response or secondary LOR in the real-world setting.

The cohort was representative of Canadian patients with IBD in real-world practice, given that participation in the Canadian support program was available to all patients prescribed commercial vedolizumab. Therefore, the resulting patient characteristics aligned with those typically reported in observational IBD studies: the median age was 48 years for patients with CD and 44 years for those with UC, and female patients comprised 570 (62%) and 930 (51%) of these groups, respectively, and all patients had moderate to severe disease.^8^^,^^9^^,^^17^

Among included patients, clinical remission rates at 6 weeks following treatment initiation were consistent with previous studies.^17^ For patients with CD, clinical remission rates were 30% at 6 weeks. For patients with UC, clinical remission rates were 47% at 6 weeks. These results are comparable to those presented in a recent systematic review and meta-analysis of real-world effectiveness of vedolizumab, which showed clinical remission rates of 24% for CD patients and 43% for UC patients at 6 weeks.^17^ Similar trends were observed for clinical response rates. These findings highlight vedolizumab’s effectiveness as a strong induction therapy for UC and CD in a real-world cohort of IBD patients. Additional confirmation is anticipated from the ongoing VOICE study (NCT06249555), which examines the induction clinical response to vedolizumab and IL-23 antagonists in CD patients.^18^

Within the first 2 years of vedolizumab treatment we observed dose escalation patterns among patients with CD, including 165/413 (39%) of bio-naïve patients dose-escalated to Q4W (104 of which were not in clinical remission) and 273/511 (54%) of bio-experienced patients dose escalated (193 of which were not in clinical remission). Among patients with UC, 489/1294 (39%) of bio-naïve patients dose-escalated to Q4W (315 of which were not in clinical remission) and 258/522 (50%) of bio-experienced patients dose escalated (166 of which were not in clinical remission). These rates of dose escalation are broadly similar to those previously reported for vedolizumab and are characteristic of biologic therapies for IBD.^9^^,^^10^ Health Canada’s vedolizumab product monograph references dose escalation to Q4W for patients with a decreased clinical response, and this practice is widely reimbursed by both private and public insurers in Canada. This results in minimal barriers to dose escalation in Canada, which may explain the slightly higher rates of dose escalation observed compared to other countries.^10^

Following Q4W dose escalation, approximately half of all patients improved and recaptured clinical response to treatment and were in either clinical response or clinical remission at 12 weeks after dose escalation. Rates of clinical remission were sustained or improved through 52 weeks, supporting real-world effectiveness of vedolizumab dose escalation in recapturing clinical response among patients who experienced suboptimal clinical response and LOR. Results are comparable to the systematic review of real-world effectiveness of dose escalation, in which 49.6% of patients with IBD had a clinical response within 54 weeks of vedolizumab dose escalation.

A strength of the study lies in its large cohort and the generalizability of the findings, as it utilized nationwide real-world data encompassing all patients receiving commercial vedolizumab. However, a potential limitation is the reliance on clinical disease scores, such as HBI and pMs, to define clinical response and clinical remission, rather than employing objective measures for disease assessment like endoscopy or biomarker normalization. However, despite this limitation, results from Afif et al. 2024 which explored faecal calprotectin (FCP) and C-reactive protein (CRP) biomarkers as indicators of clinical response/clinical remission is supportive of the week 6 clinical remission results.^19^

Additional limitations are inherent to the data available through the support program. Specifically, information regarding the rationale for dose escalation is not captured. To mitigate this constraint, the present analyses concentrated on patients with demonstrable potential for clinical improvement, as defined by their baseline HBI and pMs scores, and patients in clinical remission were not included in the analyses. Furthermore, due to the retrospective, patient-reported nature of capturing prior immunomodulator and corticosteroid use in the support program, a reliable assessment of these characteristics was not possible and are not reported in Table 1. Similarly, use of adjunct therapies during vedolizumab treatment is not collected through the PSP. The primary purpose of data collection through the program is for administrative and reimbursement requirements to support patient access; accordingly, clinical variables beyond these objectives are not collected.

Despite the limitations, this study complements findings from previous trials and studies of vedolizumab in IBD and supports the real-world effectiveness of dose escalation among vedolizumab-treated patients with CD and UC who experienced suboptimal clinical response or LOR, in recapturing clinical response.

## Supplementary Material

gwaf033_Supplementary_Data

## Data Availability

The data underlying this article cannot be shared publicly due to the privacy of individuals that participated in the nation-wide patient support program, developed by Takeda Canada Inc. The data will be shared on reasonable request to the corresponding author.
