# Supplementary material for: Effectiveness of vedolizumab dose escalation in inflammatory bowel disease in a large-scale, Canadian real-world cohort
Source: J Can Assoc Gastroenterol. 2025 Dec 9;9(1):30–7. doi: 10.1093/jcag/gwaf033 (PMC12884842; doi:10.1093/jcag/gwaf033)
Supplement: gwaf033_Supplementary_Data [file gwaf033_supplementary_data.zip › gwaf033_Supplementary_Data/Vedolizumab_Dose_Escalation_Manuscript_Supplementary_Material.docx]

**Supplementary Material – Effectiveness of Vedolizumab Dose Escalation in Inflammatory Bowel Disease in a Large-Scale, Canadian Real-World Cohort**


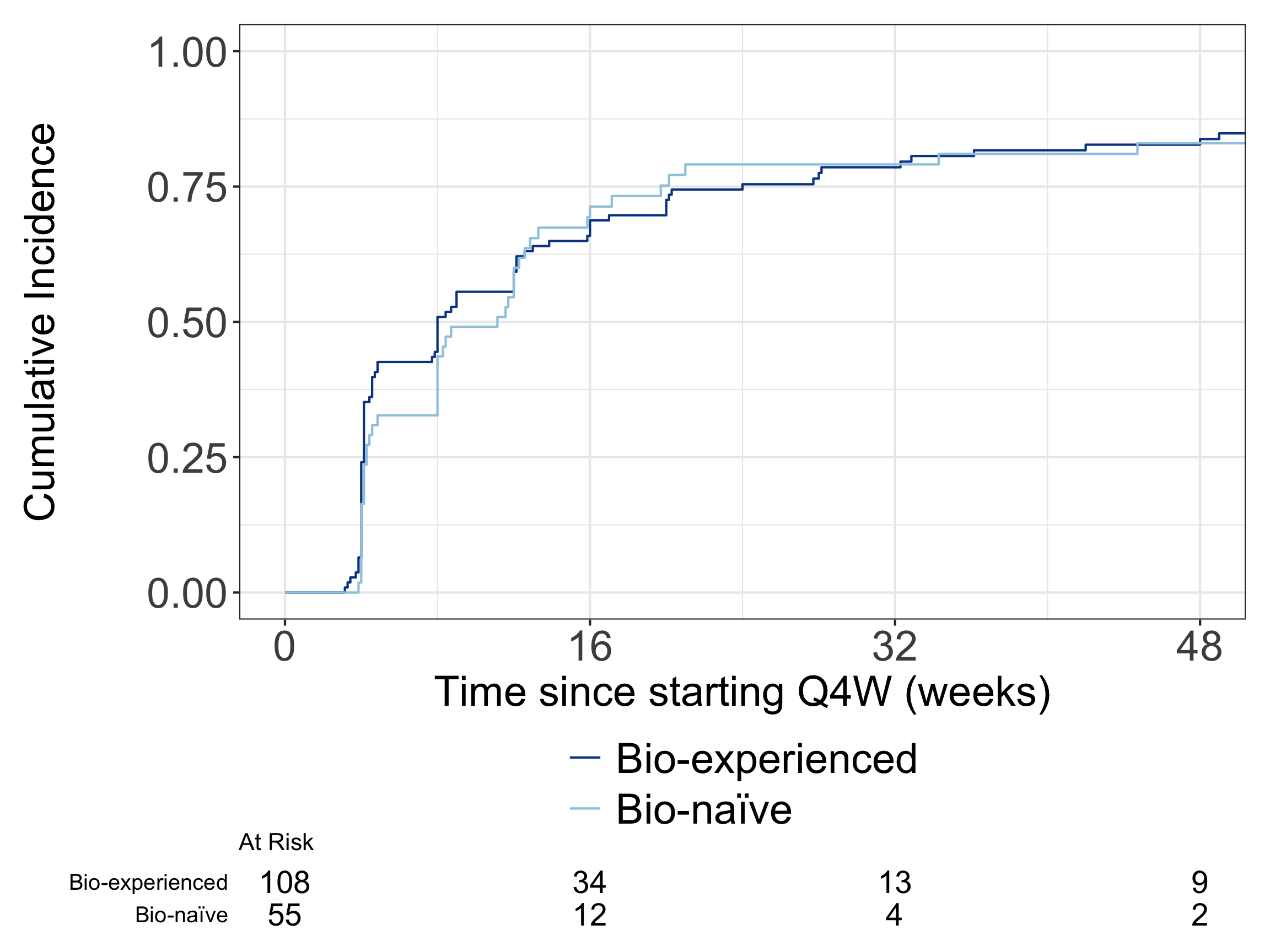


**Supplementary Figure 1A.** Time to response following Q4W dose escalation among CD patients in the Q8W>Q4W dose escalation cohort


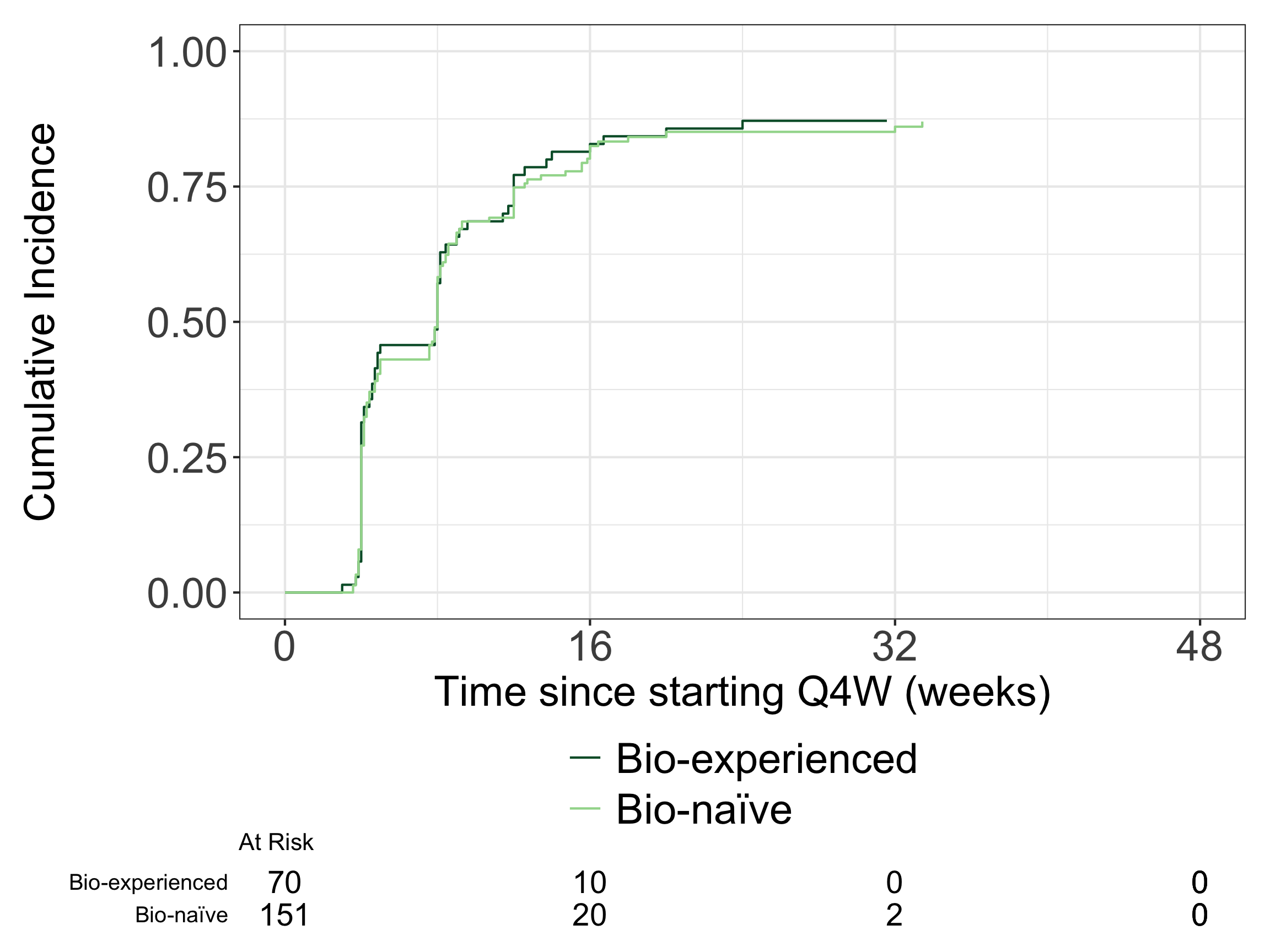


**Supplementary Figure 1B.** Time to response following Q4W dose escalation among UC patients in the Q8W >Q4W dose escalation cohort


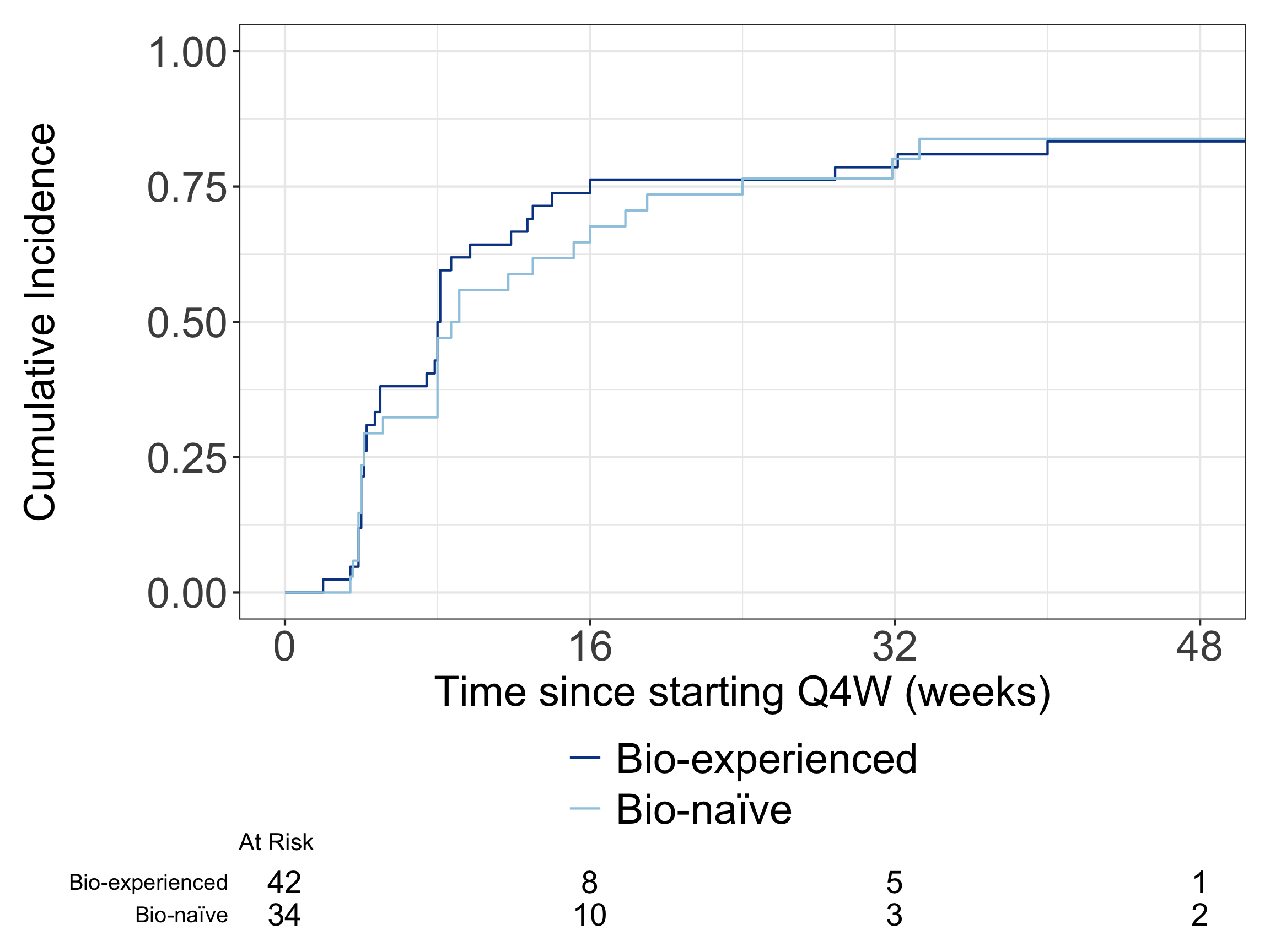


**Supplementary Figure 2A.** Time to response following Q4W dose escalation among CD patients in the Q14>Q4W dose escalation cohort


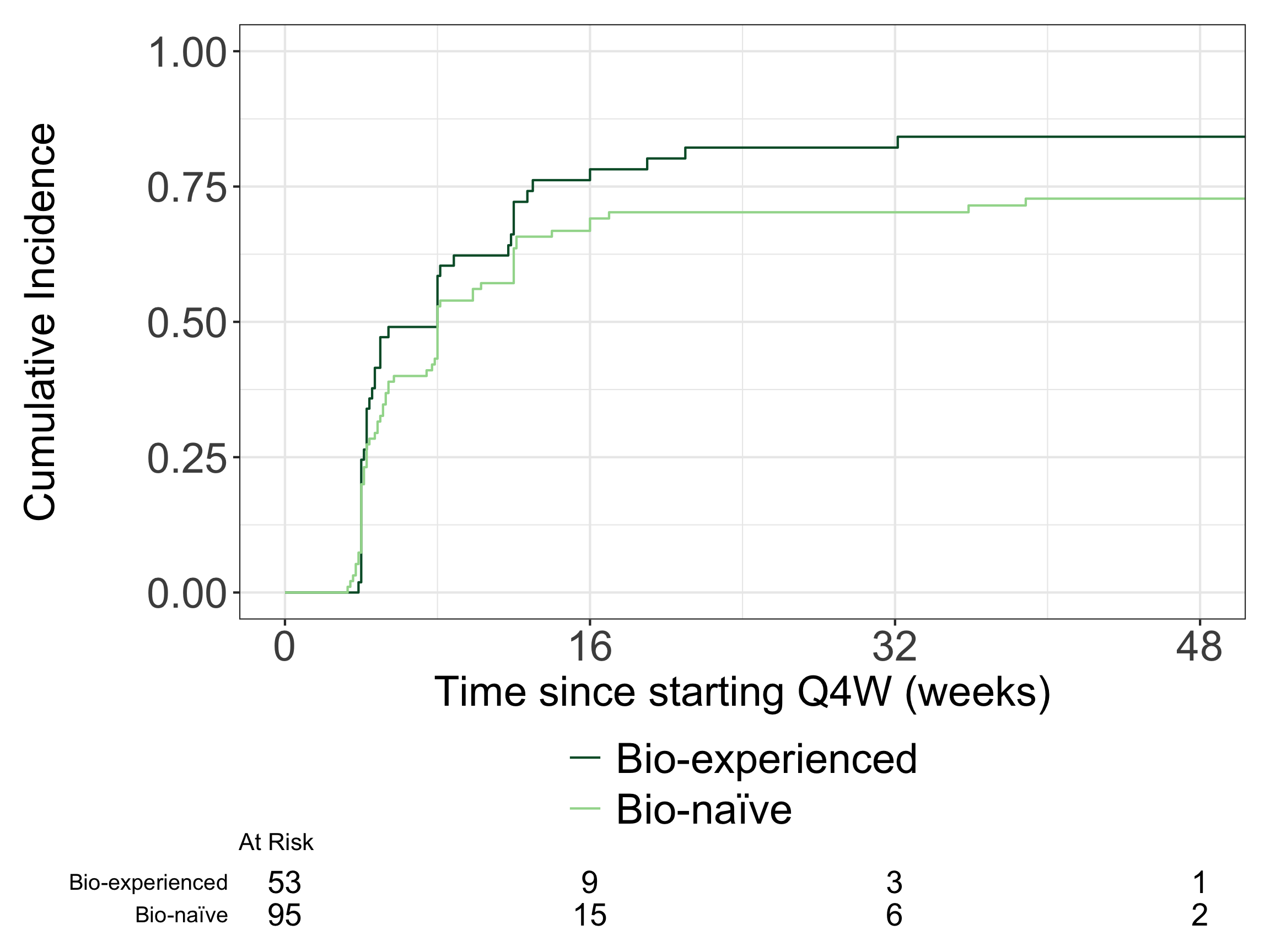


**Supplementary Figure 2B.** Time to response following Q4W dose escalation among UC patients in the Q14>Q4W dose escalation cohort


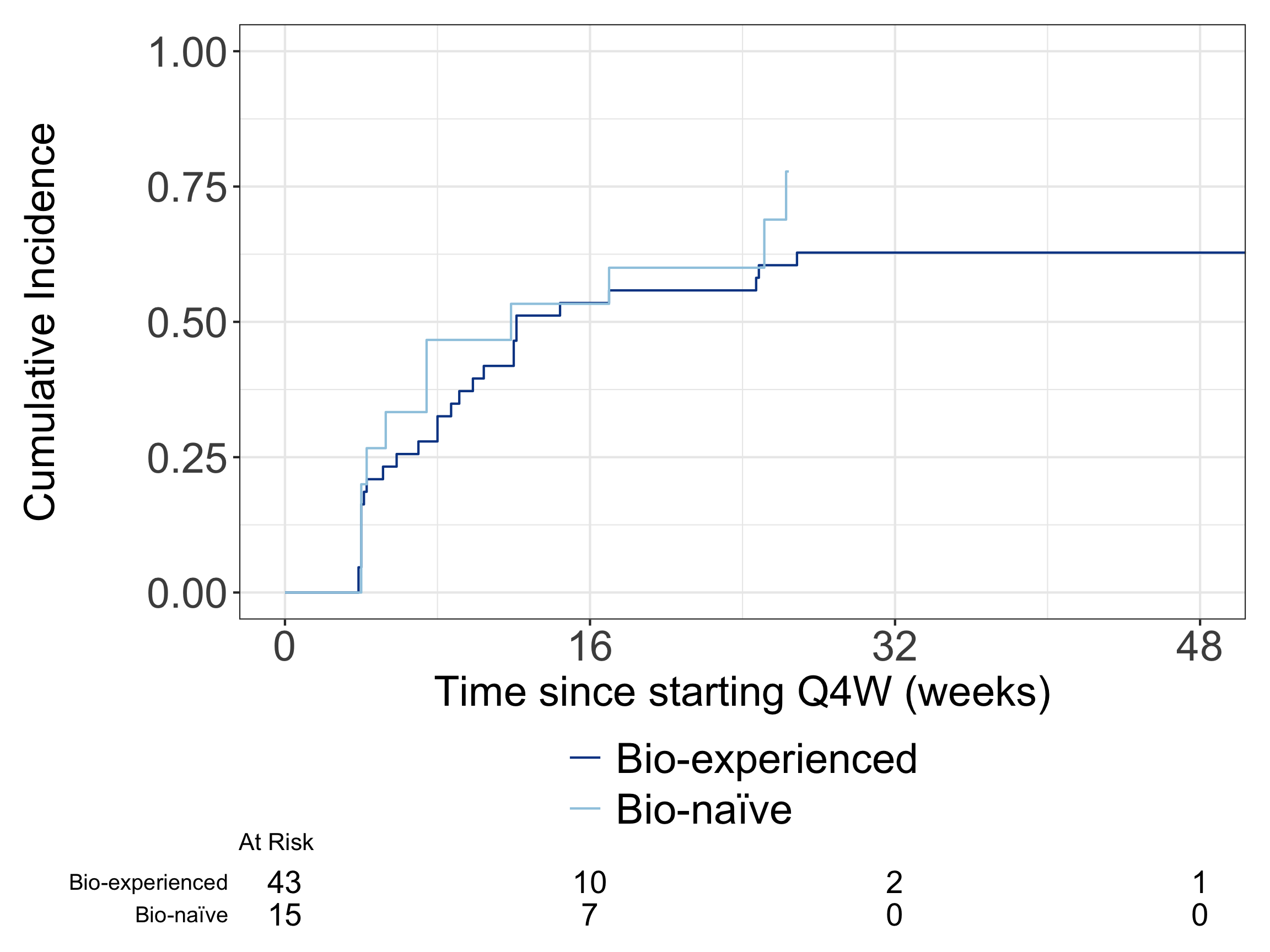


**Supplementary Figure 3A.** Time to response following Q4W dose escalation among CD patients in the Q10>Q4W dose escalation cohort


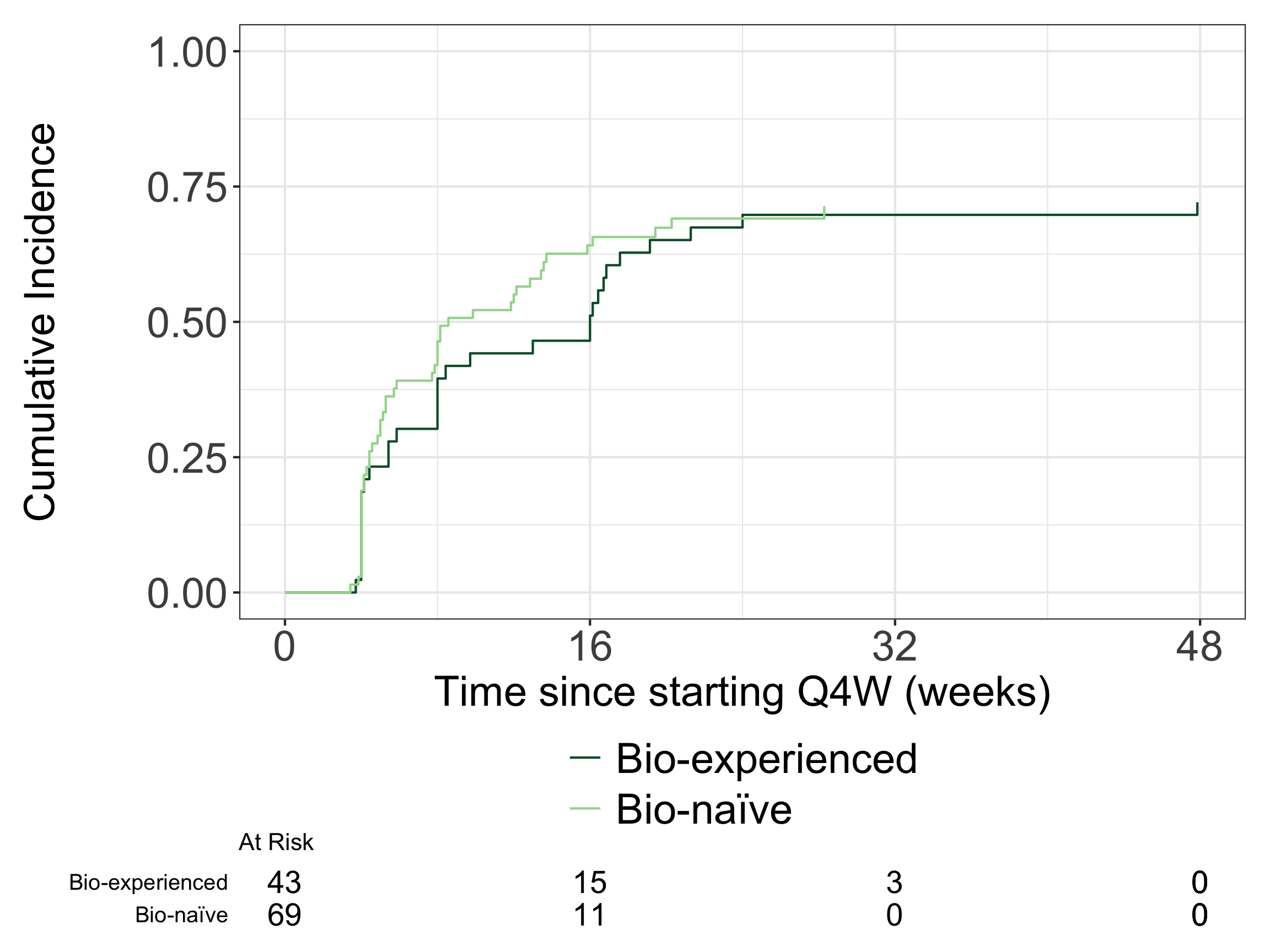


**Supplementary Figure 3B.** Time to response following Q4W dose escalation among UC patients in the Q10>Q4W dose escalation cohort

**Supplementary Table 1: Logistic regression model of remission 12 weeks after Q4W dose escalation in patients who initially respond**

| **Characteristic** | **Odds ratio** | **95% confidence interval** | **p-value** |
| --- | --- | --- | --- |
| **Primary Diagnosis** |  |  |  |
| Crohn's Disease | — | — |  |
| Ulcerative Colitis | 0.93 | 0.53, 1.64 | 0.8 |
| **Biologic exposure** |  |  |  |
| Bio-experienced | — | — |  |
| Bio-naïve | 1.44 | 0.85, 2.46 | 0.2 |
| **Age at initiation (years)** | 1.01 | 0.99, 1.02 | 0.5 |
| **Sex** |  |  |  |
| Female | — | — |  |
| Male | 1.00 | 0.61, 1.62 | >0.9 |
| **Disease duration** | 0.99 | 0.97, 1.02 | 0.6 |
| **HBI/PMS score*** |  |  |  |
| High HBI/PMS | — | — |  |
| Low HBI/PMS | 1.36 | 0.84, 2.22 | 0.2 |

* Divided based on medians of HBI and PMS
